# Antioxidant and phytochemical analysis of *Ranunculus arvensis* L. extracts

**DOI:** 10.1186/s13104-015-1228-3

**Published:** 2015-06-30

**Authors:** Muhammad Zeeshan Bhatti, Amjad Ali, Ayaz Ahmad, Asma Saeed, Salman Akbar Malik

**Affiliations:** Department of Biochemistry, Faculty of Biological Sciences, Quaid-i-Azam University, Islamabad, 45320 Pakistan; Institute of Biomedical Sciences, School of Life Science, East China Normal University, 500 Dongchuan Road, Shanghai, 200241 People’s Republic of China; Department of Biotechnology, Abdul Wali Khan University, Mardan, 23200 Pakistan; Department of Biological Sciences, Gomal University, Dera Ismail Khan, 29050 Pakistan

**Keywords:** Antioxidant, *Ranunculus arvensis*, Phenolic content, Flavonoid content, HPLC

## Abstract

**Background:**

*Ranunculus arvensis* L. (*R. arvensis*) has long been used to treat a variety of medical conditions such as arthritis, asthma, hay fever, rheumatism, psoriasis, gut diseases and rheumatic pain. Here, we screened *R. arvensis* for antioxidant activity, phytochemical and high performance liquid chromatography (HPLC) analyses.

**Methods:**

The chloroform, chloroform:methanol, methanol, methanol:acetone, acetone, methanol:water and water extracts of *R. arvensis* were examined for DPPH (1, 1-diphenyl-2-picrylhydrazyl) free radical scavenging assay, hydrogen peroxide scavenging assay, phosphomolybdenum assay, reducing power assay, flavonoid content, phenolic content and high performance liquid chromatography analysis.

**Results:**

Significant antioxidant activity was displayed by methanol extract (*IC*_50_ 34.71 ± 0.02) in DPPH free radical scavenging assay. Total flavonoids and phenolics ranged 0.96–6.0 mg/g of extract calculated as rutin equivalent and 0.48–1.43 mg/g of extract calculated as gallic acid equivalent respectively. Significant value of rutin and caffeic acid was observed via high performance liquid chromatography.

**Conclusions:**

These results showed that extracts of *R. arvensis* exhibited significant antioxidant activities. Moreover, *R. arvensis* is a rich source of rutin, flavonoids and phenolics.

## Background

*Ranunculus arvensis* L., (*R. arvensis*) belongs to the family Ranunculaceae which is commonly known as corn buttercup. It is widely used to treat arthritis, asthma, hay fever, rheumatism, psoriasis and gut diseases [1]. It is also used as poultice around the knees and thumbs for rheumatic pain [2]. The fresh plant is toxic because it contains acrid sap that can cause blistering of skin however, its toxicity is abolished when dried [1]. From the beginning of civilization, plants have been used to treat diseases, as source of food, shelter, fodder, timber, fuel, and also in health-care [3]. Many plants are widely used in traditional medicines. They contain active chemical constituents that produce therapeutic physiological effects to treat a variety of diseases in both humans and animals [4]. Natural products from medicinal plants are considered chemically balanced, effective and least harmful with minimal side effects as compared to synthetic medicines. These medicinal plants have long been effective used in both traditional and modern medicine as nutraceuticals as well as food supplements. The World Health Organization (WHO) estimated that 60–70% of the population of developing countries use medicinal plants for the treatment of ailments [5].

Certain diseases are caused by the free radicals which can cause irreversible oxidative damage to the living system [6, 7]. The oxidation induced by reactive oxygen species results in membrane protein damage and DNA mutation, which can lead to development and propagation of many diseases, such as tissue injury, cardiovascular diseases, inflammation, mutation in genetic material, cancer and human neurological disorders [8–10]. Antioxidants can protect human from these free radicals and/or delay the development of diseases caused by these free radicals [11, 12]. Synthetic antioxidants such as butylated hydroxyanisole (BHA) and butylated hydroxytoluene (BHT) have been used as antioxidant agents since the beginning of this century but these are prohibited due to their in vivo carcinogenic effects. Therefore, a significant effort has been spent to find out natural antioxidants over synthetic compounds and elimination of synthetic antioxidants [13]. Polyphenols are widely distributed in plants and play an important role in medicine. Flavonoids and phenolics are a significant constituent of the human diet and many of them are natural antioxidants [14]. These phytochemicals have wide pharmacological and biological applications and can be used to treat coronary heart diseases, cancer and mutagenesis [15].

To our knowledge, there are no reports on antioxidant activity of *R. arvensis.* The present investigation was designed to determine antioxidant activity by DPPH (2, 2-diphenyl-1-picrylhydrazyl) free radical scavenging assay, hydrogen peroxide scavenging assay, phosphomolybdenum assay, reducing power assay, phytochemical screening (total flavonoids content and total phenolics content). Moreover, we also determined the effects of different extracts by high performance liquid chromatography (HPLC) analysis.

## Methods

### Preparation of plant extracts

Fresh *R. arvensis* (L.) was collected in May 2011 from F. R. Bannu (32°56′ 33°16′ North latitudes and 70°22′ 70°52′ longitudes), located on the East of Bannu District, Khyber Pakhtunkhwa, Pakistan. Taxonomic identification of the plant was done by taxonomist Department of Plant Sciences, Quaid-i-Azam University, Islamabad, 45320, Pakistan and Department of Botany, Government Post Graduate College, Bannu, Pakistan. The voucher specimen (AR-57) was deposited in the herbarium. The plant was rinsed with distilled water and shade dried. The extracts were prepared by soaking 30 g of ground plant powder in 300 mL of various solvents i.e. chloroform, chloroform:methanol (1:1), methanol, methanol:acetone (1:1), acetone, methanol:water (1:1) and water. They were placed in a shaking incubator (1575-2, Shel Lab., USA) at 150 rpm for 24 h at room temperature (28 ± 2°C) and sonicated for 5 min after 12 h. It was filtered with Whatmann No. 41 filter paper and concentrated in rotary evaporator (BUHI Rotavapor R-20, Switzerland) at 40°C. Fully dried extracts were packed in seal-pack containers and stored at −20°C for further experiments.

### Chemicals

Aluminum chloride, ammonium molybdate, ascorbic acid (Vitamin-C), caffeic acid, catechin, dibasic sodium phosphate, 1, 1-diphenyl-2-picrylhydrazyl (DPPH), ferric chloride, Folin–Ciocalteu reagent, gallic acid, hydrogen chloride, hydrogen peroxide (H_2_O_2_), kaempferol, monobasic sodium phosphate, myrecitin, nitric acid, potassium acetate, potassium ferricyanide, quercetin, rutin sodium carbonate, sodium phosphate, trichloroacetic acid, chloroform, methanol, acetone, and dimethyl sulphoxide (DMSO) were purchased from Sigma-Aldrich chemical co.

### DPPH (2, 2-diphenyl-1-picrylhydrazyl) free radical scavenging assay

The free radical scavenging potential of different extracts were determined according to the procedure of Kulisic with some modifications [16]. An aliquot of 50 µL of sample solution of various concentrations (25–400 μg/mL) were mixed with 950 µL of methanolic solution of DPPH (3.4 mg/100 mL). The reaction mixture was incubated at 37°C for 1 h in the dark. The free radical scavenging potential of the extracts were expressed as the disappearance of the initial purple color. The absorbance of the reaction mixture was recorded at 517 nm using UV–Visible spectrophotometer (Agilent 8453, Germany). Ascorbic acid was used as the positive control. DPPH scavenging capacity was calculated by using the following formula:

$${\text{Scavenging}}\;{\text{activity}}\;(\% ) = \left( {\frac{{{\text{Absorbance}}^{\text{control}} - {\text{Absorbance}}^{\text{sample}} }}{{{\text{Absorbance}}^{\text{control}} }}} \right)\; \times \;100$$

### Hydrogen peroxide scavenging assay

The ability of the extract to scavenge hydrogen peroxide (H_2_O_2_) was determined according to the method of Ruch et al. [17]. Aliquot of 0.1 mL of extracts (25–400 μg/mL) was transferred into the eppendorf tubes and their volume was made up to 0.4 mL with 50 mM phosphate buffer (pH 7.4) followed by the addition of 0.6 mL of H_2_O_2_ solution (2 mM). The reaction mixture was vortexed and after 10 min of reaction time, its absorbance was measured at 230 nm. Ascorbic acid was used as the positive control. The ability of the extracts to scavenge the H_2_O_2_ was calculated using the following equation:

$${\text{H}}_{2} {\text{O}}_{2} \;{\text{scavenging}}\;{\text{activity}}\;{\text{percentage}} = [(A_{0} - A_{1} )/A_{0} ] \, \times \, 100$$

where: A_0_ = Absorbance of control, A_1_ = Absorbance of sample.

### Phosphomolybdenum assay

For the conduction of the phosphomolybdenum assay, the method of Prieto et al. was followed [18]. An aliquot of 0.1 mL of sample solution of different concentrations (25–400 μg/mL) treated with 1 mL of reagent solution (0.6 M sulfuric acid, 28 mM sodium phosphate and 4 mM ammonium molybdate). The tubes were incubated at 95°C in a water bath for 90 min. The samples were cooled to room temperature and their absorbance was recorded at 765 nm. Ascorbic acid was used as the positive control. Antioxidant capacity was estimated by using following equation:

$${\text{Antioxidant}}\;{\text{activity }}\% \; = \;[({\text{Absorbance control }} - {\text{ Absorbance sample}})/{\text{Absorbance control}}] \, \times \, 100.$$

### Reducing power assay

The reducing power was determined according to the Oyaizu et al. method with some modifications [19]. Aliquot of 0.2 mL of various concentrations of the extracts (25–400 μg/mL) were mixed separately with 0.5 mL of phosphate buffer (0.2 M, pH 6.6) and 0.5 mL of 1% potassium ferricyanide. The mixture was incubated in a water bath at 50°C for 20 min. After cooling at room temperature, 0.5 mL of 10% trichloroacetic acid was added to it followed by centrifugation at 3,000 rpm for 10 min. Supernatant (0.5 mL) was collected and mixed with 0.5 mL of distilled water. Ferric chloride (0.1 mL of 0.1%) was added to it and the mixture was left at room temperature for 10 min. The absorbance was measured at 700 nm. Ascorbic acid was used as positive control.

### Determination of total flavonoid content

The total flavonoid content was determined by the aluminum chloride colorimetric method as described by Chang et al. with some modifications [20]. Aliquot of 0.5 mL of various extracts (1 mg/mL) were mixed with 1.5 mL of methanol, followed by the addition of 0.1 mL of 10% aluminum chloride, 0.1 mL of potassium acetate (1 M) and 2.8 mL of distilled water. The reaction mixture was kept at room temperature for 30 min. Absorbance of the reaction mixture was recorded at 415 nm. The calibration curve (0–8 µg/mL) was plotted using rutin as a standard. The total flavonoids were expressed as mg of rutin equivalent/gram dry weight.

### Determination of total phenolic content

The amount of total phenolic content was determined according to the Velioglu method using the Folin–Ciocalteu reagent [21]. Aliquot of 0.1 mL of various extracts (4 mg/mL) was mixed with 0.75 mL of Folin–Ciocalteu reagent (10-fold diluted with dH_2_O). The mixture was kept at room temperature for 5 min and 0.75 mL of 6% sodium carbonate was added. After 90 min of reaction, its absorbance was recorded at 725 nm. The standard calibration (0–25 μg/mL) curve was plotted using gallic acid. The total phenolics were expressed as mg gallic acid equivalent/gram dry weight. Negative control was prepared by adding 0.1 mL of DMSO instead of extract.

### High performance liquid chromatography analysis

For the analysis of flavonoids and phenolics, stock solutions of caffeic acid, catechin, kaempferol, myricetin, rutin, quercetin and gallic acid were prepared in methanol (1 mg/mL). Solutions were filtered by 0.2 µm Sartolon Polyamide membrane filter (Sartorius). The calibration curve was raised by 10, 20, 50, 100, 150 and 200 µg/mL. The crude extracts of *R. arvensis* were prepared at concentration of 10 mg/mL in methanol. The extracts were dissolved in methanol with the aid of sonication and were filtered through 0.2 µm Sartolon Polyamide membrane filter (Sartorius). All the samples were prepared fresh and used for analysis immediately.

The analysis was carried out by using Agilent Chem. station Rev.B.02-01-SR1(260) software and Agilent 1200 series binary gradient pump coupled with a diode array detector (DAD; Agilent technologies, Germany) having Discovery-C18 analytical column (4.6 × 250 mm, 5 µm particle size, Supelco, USA). Method followed was as described by Zu et al. with slight modification according to the system suitability [22]. Briefly, mobile phase-A was methanol:acetonitrile:water:aectic acid (10:5:85:1) and mobile phase B was methanol:acetonitrile:acetic acid (60:40:1). A gradient of time 0–20 min for 0–50% B, 20–25 min 50–100% B and then isocratic 100% B till 30 min was used. Flow rate was 1 mL/min and injection volume was 20 µL. Rutin and gallic acid were analyzed at 257 nm, catechin at 279 nm, caffeic acid at 325 nm and quercetin, myricetin, kampferol was analyzed at 368 nm. Each time the column was preconditioned for 10 min before the next analysis.

### Statistical analysis

Results were expressed as mean ± standard deviation of three replicates. CoStat statistical program 6.400^®^ (2008©, USA) was used for statistical analysis. Analysis of variance (ANOVA) was performed through Bartlett’s Test. Latin square design (LSD) was applied to testify the significance of concentrations and extracts.

## Results and discussion

### DPPH (2, 2-diphenyl-1-picrylhydrazyl) free radical scavenging assay

**Figure 1:**
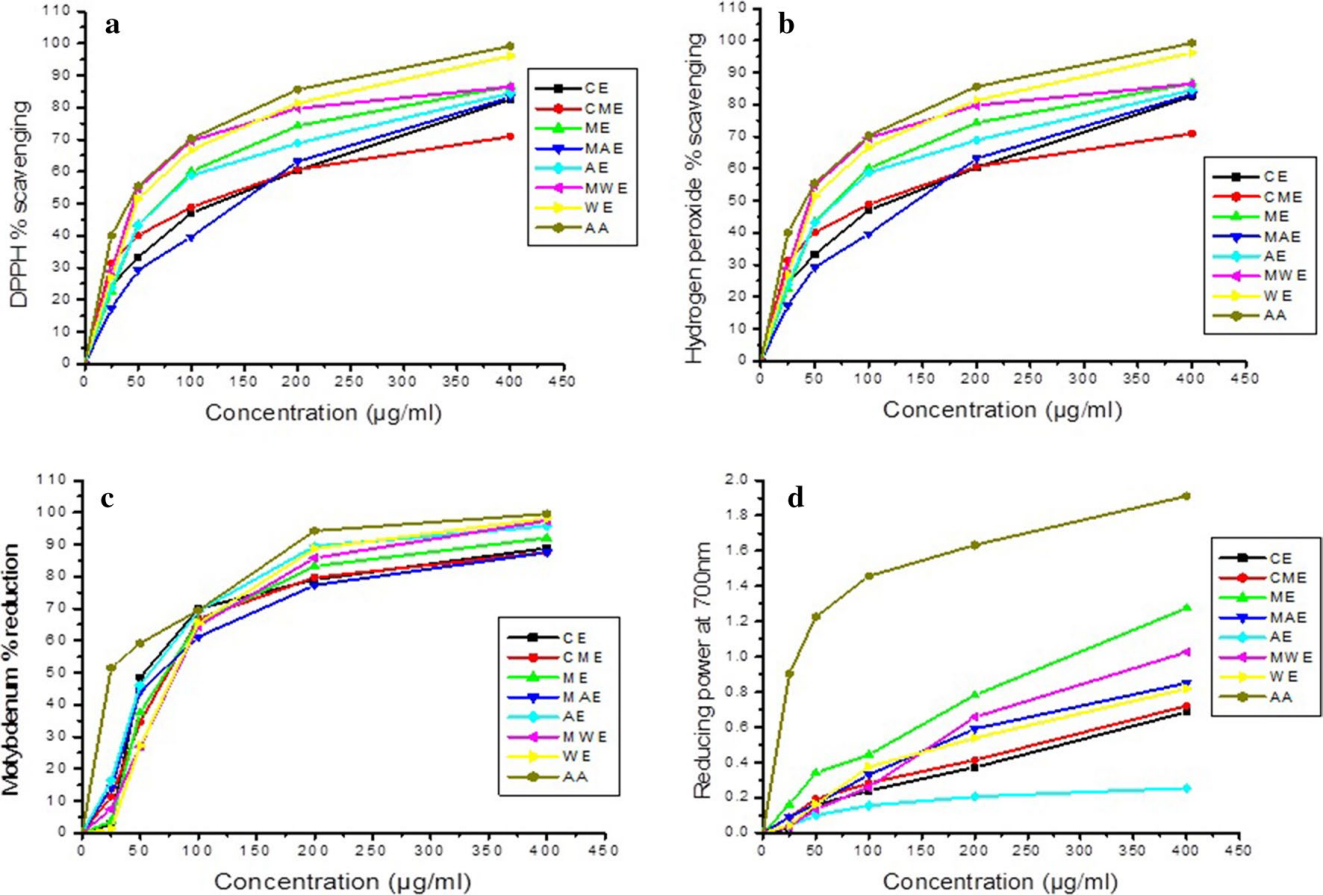
Antioxidant activities of *R. arvensis* extracts: **a** DPPH free radical scavenging activity; **b** hydrogen peroxide scavenging activity; **c** motybdenum ion percentage reduction; **d** reducing capacity.

The antioxidant activity of different extracts of *R. arvensis* was primarily assessed by 2, 2-diphenyl-1-picrylhydrazyl (DPPH), which is based on the ability of DPPH to react with proton donors such as phenols. The other members of family Ranunculaceae were previously assessed for free radical scavenging by many groups. However, *R. arvensis* free radical scavenging ability remains unknown. We showed that *R. arvensis* exhibits significant free radical scavenging potential especially its methanol extract (*IC*_*50*_: 34.71 µg/mL; Table 1). The percentages of free radical scavenging are given in Figure 1. The DPPH activity demonstrated in *Nigella sativa* was EC_50_ (29.40 ± 0.35) [23], while *IC*_*50*_ values of chloroform extract and ethyl acetate extract were 106.56 and 121.62 μg/mL, respectively [24]. Zengin et al. reported *IC*_*50*_ value of crude extract of *Centaurea urvillei was* 137.06 μg/mL [25]. These results show that *R. arvensis* is a good source for DPPH free radical scavenging activity as compared to the other members of the family.

**Table 1:** IC_50_ values of various extracts of *R. arvensis*

Antioxidant assays			
IC_50_ (µg/mL)			
Extract	DPPH free radical scavenging assay	Hydrogen peroxide scavenging assay	Phosphomolybdenum assay
Chloroform extract	330.29 ± 0.01	124.36 ± 0.01	52.58 ± 0.01
Chloroform:methaol extract	186.28 ± 0.01	101.6 ± 0.01	69.39 ± 0.03
Methanol extract	34.71 ± 0.02	65.73 ± 0.01	66.06 ± 0.01
Methanol:acetone extract	285.28 ± 0.01	134.68 ± 0.01	63.09 ± 0.01
Acetone extract	264.08 ± 0.01	69.55 ± 0.01	56.29 ± 0.01
Methanol:water extract	47.61 ± 0.02	43.53 ± 0.02	77.95 ± 0.01
Water extract	85.11 ± 0.02	51.27 ± 0.01	74.37 ± 0.01
Ascorbic acid^a^	6.38 ± 0.01	39.05 ± 0.01	26.16 ± 0.01
LSD value	8.14	6.30	9.90
CV	12.01%	8.49%	12.72%
R^2^	0.96	0.97	0.96

*p* < 0.05.

*LSD* least significant difference, *CV* coefficient of variation, *LD*_*50*_ lethal dose, 50%, *IC*_*50*_ half maximal inhibitory concentration.

^a^Positive control values are expressed as ascorbic acid (AA) (average ± SD; n = 4).

### Hydrogen peroxide scavenging assay

The scavenging effect of different extracts of *R. arvensis* on hydrogen peroxide was concentration-dependent (25–400 μg/mL) as shown in Figure 1 (*P* < 0.05). The methanol:water extract displayed strong H_2_O_2_ scavenging activity (*IC*_*50*_ 43.53 µg/mL). whereas water extract exhibited *IC*_*50*_ 51.27 µg/mL (Table 1). The significant difference in percentage inhibition of H_2_O_2_ of all extracts was compromising in Figure 1*P* < 0.05. Among various plants of the Ranunculaceae, *Gymnema sylvestre* exhibited better H_2_O_2_ scavenging activity (*IC*_*50*_ 72.55 μg/mL) but comparatively less than *R. arvensis* [26]. Moreover, *Spondias pinnata* plant extract acquires *IC*_*50*_ 44.74 ± 25.61 mg/mL on the scavenging of H_2_O_2_ [27]. The naturally occurring of H_2_O_2_ in the air, water, human body, plants, microorganisms and food is at low concentration levels. It is quickly decomposed into oxygen (O_2_) and water (H_2_O) and may create hydroxyl radicals (OH) that can initiate lipid peroxidation and cause DNA damage. methanol:water extract of *R. arvensis* capably scavenged hydrogen peroxide which may be attributed to the presence of phenolic groups that could donate electrons to hydrogen peroxide, thereby neutralizing it into H_2_O.

### Phosphomolybdenum assay

This assay is based on the reduction of Mo^VI^ into Mo^V^ by a reductant with the formation of a green phosphate–Mo^V^ complex, which shows an absorbance maximum at 695 nm. The antioxidant activity of almost all extracts was not significantly different. Chloroform extract showed best total antioxidant capacity i.e. *IC*_*50*_ value was 52.58 µg/mL (Table 1). Other extracts also exhibited better *IC*_*50*_ value and molybdenum ions percentage reduction at *P* < 0.05 (Figure 1). The total antioxidant capacity of *Centaurea urvillei* with ascorbic acid (39.70 mg AE/g extract) and trolox equivalents (143.53 mg TE/g extract) [25]. *Nigella sativa* also expressed better activity in this assay (TEAC 36.38 ± 1.08) [23]. However, these findings are not comparable due to difference in solvents, measuring techniques and growth conditions.

### Reducing power assay

In this assay, the presence of e^−^-donating compounds resulted in the reduction of Fe^3+^ (ferricyanide) into Fe^2+^ (ferrous). The results are shown in the Figure 1. The reducing potential of the extracts measured for the concentration up to 400 μg/mL showed a general increase in activity when the concentration was increased. Among the tested extracts, the methanol:water extract possessed the highest reducing capacity of free radicals scavenging (1.28 ± 0.05), with absorbance at 700 nm. The extracts had better free radical reductive ability with increasing concentrations of the extract. Hazra et al. [27] reported the same behavior in *Spondias pinnata* extracts. This concentration-dependent activity pattern was also followed by *Consolida orientalis* extracts which behaved the best at 800 μg/mL [26].

### Determination of total flavonoids content

Quantitative total flavonoid determination was performed by precipitating the extracts with aluminum chloride have an intense yellow fluorescence when observed by UV spectrophotometer. Total flavonoids content were expressed as mg rutin equivalent (RE) per gram dry extract weight. Among the studied *R. arvensis* extracts, total flavonoid contents estimation revealed the presence of flavonoids, except in the chloroform extract. Significant amount of flavonoids were present in the methanol extract (6.00 ± 0.02 mg RE/g; Table 2), while comparative amount was present in methanol:water extract (5.72 ± 0.01 mg RE/g) and in the water extract (2.19 ± 0.01 mg RE/g). Previous study has shown that flavonoids were present in *R. arvensis* by the change of sample colour [28]. Hussain et al. used the titration method for identification of flavonoids in *R. arvensis* (1.769 mg/100 g) [29]. This difference may be due to different geographical distribution of the plant or changes in methodology.

**Table 2:** Identification and quantification of flavonoids and phenolics of seven crude extracts of *R. arvensis* through spectrophotometry and high performance liquid chromatography

Total flavonoids and total phenolics ± SD			HPLC profile			
Extract	mg RE/g dry extract	GAE/g dry extract	RT (min)	λ max (nm)	Compound	% of dry weight
Chloroform extract	–	–	–		–	–
Chloroform:methaol extract	1.95 ± 0.01	–	–		–	–
Methanol extract	6.00 ± 0.02	0.48 ± 0.03	15.00	257	Rutin	0.44
	–	–	10.79	325	Caffeic acid	0.017
Methanol:acetone extract	1.08 ± 0.01	–	–		–	–
Acetone extract	0.96 ± 0.01	–	–		–	–
Methanol:water extract	5.72 ± 0.01	1.06 ± 0.02	15.02	257	Rutin	0.01
Water extract	2.19 ± 0.01	1.43 ± 0.01	10.68	325	Caffeic acid	0.008

*SD* ±standard deviation, *RT* retention time.

### Determination of total phenolics content

The quantitative determination of total phenolic was carried out using Folin–Ciocalteu reagent in terms of gallic acid equivalent. Total phenolic content is expressed as mg gallic acid equivalent per gram dry extract weight. There is variation in total phenolics present in *R. arvensis* ranging from 0.48 to 1.43 mg of the total GAE/g of extract. The highest amount was shown by water extract (1.43 mg/g GAE), whereas the chloroform extract, chloroform:methanol extract, methanol:acetone extract and acetone extract remained insignificant (Table 2). Our results are more significant than the results of Hachelaf et al. which detected the presence of phenolic acid in *R. arvensis* by the change of sample color [28]. Hussain et al. found phenolic contents (0.848 mg/100 g) in *R. arvensis* using titration method [29]; the same work was performed in two other species of Ranunculus, with the highest phenolics were found in the ethyl acetate extract of *R. marginatus* (131.7 ± 4.2 mg/g GAE) and *R. sprunerianus* (140.2 ± 5.3 mg/g GAE) [30], which are comparable with our results of *R. arvensis*.

### High performance liquid chromatography analysis

**Figure 2:**
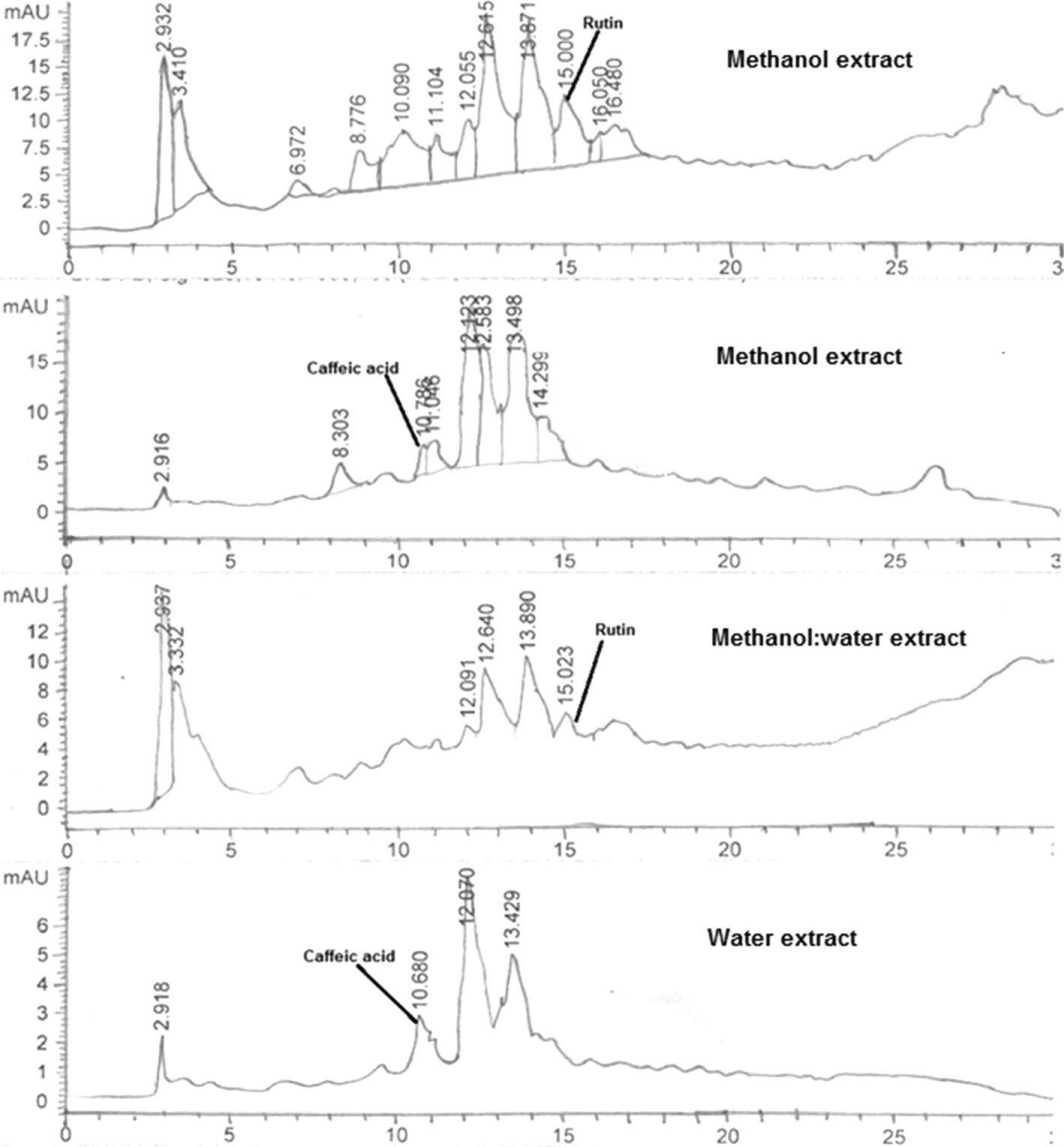
High performance liquid chromatography chromatograms of the flavonoids present in different extracts of *R. arvensis*.

The crude extracts of *R. arvensis* were assessed via seven standards (caffeic acid, catechin, kaempferol, myricetin, rutin, quercetin and gallic acid) of flavonoids and phenolics to monitor their efficiency. The HPLC profile of methanol extract of *R. arvensis* showed the presence of rutin (0.44%) and caffeic acids (0.017%). In comparison with methanol extract, smaller amount of rutin (0.01%) in methanol:water extract and caffeic acid (0.008%) in water extract (Figure 2; Table 2). The compounds belonging to classes of flavonoid and phenolics (flavonol glycosides of quercetin, kaempferol, isorhamnetin and their aglycons) were previously identified in another species of Ranunculus, *R. sardous* [31]. Previous studies showed the presence of quercetin-7-*O*-glucoside and rutin in *R. peltatus* extracts [32]. Noor et al. reported many flavonoids and phenolics from *R. repens* [33]. The presence of rutin in high quantities can be closely related to the lowest values of *IC*_*50*_ obtained for methanol extract in the DPPH assay.

## Conclusion

To the best of our knowledge, this study provides new scientific information about *R. arvensis* based on the phytochemical analysis, antioxidant potential and HPLC analysis. The various extracts *R. arvensis* showed different potential of antioxidant activity in variety of antioxidant assays. Quantitative and qualitative analysis of various crude extracts indicated the presence of bioactive compounds as flavonoids and phenolics. Moreover, the above data indicate that, *R. arvensis* was also rich in rutin and caffeic acid. However, further studies are needed for the isolation of the natural products with fascinating biological and pharmacological properties.

## Abbreviations

CE: chloroform extract
CME: chloroform:methaol extract
ME: methanol extract
MAE: methanol:acetone extract (MAE)
AE: acetone extract (AE)
MWE: methanol:water extract
WE: water extract (WE)
